# Technical Note: Ontology‐guided radiomics analysis workflow (O‐RAW)

**DOI:** 10.1002/mp.13844

**Published:** 2019-10-25

**Authors:** Zhenwei Shi, Alberto Traverso, Johan van Soest, Andre Dekker, Leonard Wee

**Affiliations:** ^1^ Department of Radiation Oncology (MAASTRO) GROW – School for Oncology and Development Biology Maastricht University Medical Centre+ Maastricht 6229 ET The Netherlands

**Keywords:** FAIR data, ontology, radiomics, semantic web, software

## Abstract

**Purpose:**

Radiomics is the process to automate tumor feature extraction from medical images. This has shown potential for quantifying the tumor phenotype and predicting treatment response. The three major challenges of radiomics research and clinical adoption are: (a) lack of standardized methodology for radiomics analyses, (b) lack of a universal lexicon to denote features that are semantically equivalent, and (c) lists of feature values alone do not sufficiently capture the details of feature extraction that might nonetheless strongly affect feature values (e.g. image normalization or interpolation parameters). These barriers hamper multicenter validation studies applying subtly different imaging protocols, preprocessing steps and radiomics software. We propose an open‐source ontology‐guided radiomics analysis workflow (O‐RAW) to address the above challenges in the following manner: (a) distributing a free and open‐source software package for radiomics analysis, (b) deploying a standard lexicon to uniquely describe features in common usage and (c) provide methods to publish radiomic features as a semantically interoperable data graph object complying to FAIR (findable accessible interoperable reusable) data principles.

**Methods:**

O‐RAW was developed in Python, and has three major modules using open‐source component libraries (PyRadiomics Extension and PyRadiomics). First, PyRadiomics Extension takes standard DICOM‐RT (Radiotherapy) input objects (i.e. a DICOM series and an RTSTRUCT file) and parses them as arrays of voxel intensities and a binary mask corresponding to a volume of interest (VOI). Next, these arrays are passed into PyRadiomics, which performs the feature extraction procedure and returns a Python dictionary object. Lastly, PyRadiomics Extension parses this dictionary as a W3C‐compliant Semantic Web “triple store” (i.e., list of subject‐predicate‐object statements) with relevant semantic meta‐labels drawn from the radiation oncology ontology and radiomics ontology. The output can be published on an SPARQL endpoint, and can be remotely examined via SPARQL queries or to a comma separated file for further analysis.

**Results:**

We showed that O‐RAW executed efficiently on four datasets with different modalities, RIDER (CT), MMD (CT), CROSS (PET) and THUNDER (MR). The test was performed on an HP laptop running Windows 7 operating system and 8GB RAM on which we noted execution time including DICOM images and associated RTSTRUCT matching, binary mask conversion of a single VOI, batch‐processing of feature extraction (105 basic features in PyRadiomics), and the conversion to an resource description framework (RDF) object. The results were (RIDER) 407.3, (MMD) 123.5, (CROSS) 513.2 and (THUNDER) 128.9 s for a single VOI. In addition, we demonstrated a use case, taking images from a public repository and publishing the radiomics results as FAIR data in this study on http://www.radiomics.org. Finally, we provided a practical instance to show how a user could query radiomic features and track the calculation details based on the RDF graph object created by O‐RAW via a simple SPARQL query.

**Conclusions:**

We implemented O‐RAW for FAIR radiomics analysis, and successfully published radiomic features from DICOM‐RT objects as semantic web triples. Its practicability and flexibility can greatly increase the development of radiomics research and ease transfer to clinical practice.

## 1 Introduction

Imaging has developed rapidly in the healthcare field and is commonly used in clinical practice. When integrated into clinical decision support systems (CDSS), 1 medical imaging could play a key role in precision medicine^2^ that could lead to better customized healthcare at an individual patient level. Multimodality medical imaging is routinely used in clinical practice, and plays a critical role in how doctors diagnose and treat cancer.

With rising utilization of imaging technology, there has been an increasing interest in the use of quantitative tumor markers derived from imaging data. Radiomics^3^, ^4^, ^5^ is an important development in quantitative imaging analysis, where digitally encoded medical images containing information related to tumor pathophysiology are converted into high‐dimensional mineable features.^5^, ^6^ Radiomics requires a high‐throughput computerized tumor feature extraction process that can operate on vast quantities of digital imaging data. The features extracted from imaging data have been associated with key clinical outcomes (e.g., overall survival). Previous studies^7^, ^8^, ^9^, ^10^, ^11^ have shown the value of radiomics on quantifying the tumor phenotype and predicting treatment response in clinical settings. By developing diagnostic and prognostic signatures, radiomics is expected to provide additional and complementary information to clinical factors for decision support.

However, three major challenges impede the pace of radiomics research and its clinical adoption: (a) lack of standardized methodology for radiomics analyses; (b) insufficient information in the feature lexicon to fully characterize the preprocessing steps leading up to feature extraction; and (c) insufficient information in the extracted feature values for an independent investigator to reproduce the same values (such as image normalization or interpolation parameters). These issues above hamper multicenter studies because of subtly different imaging protocols, preprocessing steps and extraction software. As a result, the development of radiomics research has been impeded. There is a need for an open‐source package to make radiomic features more readily comparable for researchers and clinical users. We hypothesize that comparative research will be supported if we not only share radiomic features values, but also information about the preprocessing and computational steps that led to that specific feature value.

An option to address the sharing problem is to progressively lengthen a human‐readable label as the feature name, for example *log.sigma.3.0.mm.3D_firstorder_Kurtosis*, but this can become unwieldy if the complexity of metadata increases. An alternative is to provide comprehensive dictionaries so that feature definitions can be cross‐referenced, however this also becomes cumbersome when multiple software packages, imaging settings and processing steps come into play. The Semantic Web approach has added value here, since each calculated value of a feature can be defined with a unique identifier independently of its human‐readable feature name and additional unique identifiers can be attached which acts as metadata describing that feature. For the purpose of easy comparison, sharing and validation of radiomic results, a feasible approach is to build FAIR (findable, accessible, interoperable, reusable)^12^ radiomic data via an open and extensible semantic ontology for annotating radiomic feature values with metadata and unique identifiers.

In this article, we propose an open‐source ontology‐guided radiomics analysis workflow (O‐RAW) to address the above challenges in the following manner: (a) distributing a free and open‐source software package for radiomics analysis, (b) using a domain‐specific semantic web ontology to uniquely describe features in common usage, and (c) providing methods to publish radiomic features as a semantically‐interoperable data graph object complying to FAIR data principles. With this resource, we aim to support further standardization radiomics analysis with the use of ontologies, promote multicenter collaboration via a novel learning approach using Semantic web [i.e., resource description framework (RDF)]^13^, ^14^, ^15^ and hence increase the potential for wide external validation and validity of radiomics‐assisted clinical prediction models.

## 2 Materials and Methods

### 2.1 Datasets

Imaging data from different modalities (i.e., CT, PET and MRI) were used in this study:

1. RIDER test‐retest dataset^16^: 31 sets of lung tumor CT scans with associated RTSTRUCT;
2. Multidelineation (MMD) dataset^17^: 21 sets of lung tumor CT scans and corresponding RTSTRUCT with manual delineations from five different oncologists;
3. CROSS trial dataset^18^: 79 sets of esophageal tumor PET scans and corresponding RTSTRUCT.
4. THUNDER trial dataset: 23 apparent diffusion coefficient (ADC) maps in locally advanced rectal cancer patients. Corresponding RTSTRUCT was delineated manually by three different observers.

Of the above, the RIDER and MMD datasets are publically available via an image repository (http://xnat.bmia.nl).

### 2.2 O‐RAW architecture

The O‐RAW (version 2.0) workflow package (https://gitlab.com/UM-CDS/o-raw) was developed using the Python programming language, which encapsulates the workflow in three major steps and uses two open‐source component packages (PyRadiomics^19^ as radiomics feature extractor and PyRadiomics Extension^20^). PyRadiomics is an open‐source package for radiomics extraction, which can be applied on both two and three‐dimensional medical imaging. The primary goal of PyRadiomics is to build an open‐source platform that could provide standardized methods for easy and reproducible radiomics extraction and analysis. To achieve this goal, four steps are applied in PyRadiomics: (a) loading and pre‐processing of scans and associated segmentation; (b) application of enabled filters; (c) radiomic features extraction from different classes; and (d) returning a Python dictionary object containing, configuration information, feature names and values. There are several available open‐source radiomics software such as MITK,^21^ Mazda,^22^ PyRadiomics,^19^ IBEX^23^ and CERR.^24^ Apte et al.,^24^ described the main characteristics including limitations of some software (Table S1). The current limitations of PyRadiomics are partially solved by O‐RAW, such as (a) directly take original DICOM images and RTSTRUCT files as input; (b) describing the process of feature extraction by a universal lexicon (i.e., an ontology) rather than literal expressions. The PyRadiomics Extension package aims to extend the functionality of PyRadiomics on both the input and output sides and allows users to employ native DICOM series and RTSTRUCT directly for radiomics extraction, and convert the radiomic features (Python dictionary object) to RDF using the relevant semantic ontology (i.e., Radiomics Ontology^25^). The conversion is done by mapping individual radiomic feature of PyRadiomics to unique identifiers defined by the image biomarker standardisation initiative (IBSI). If features do not exist or do not match with the IBSI identifiers, these are defined and labeled using the domain‐specific Radiomics Ontology.

**Figure 1:**
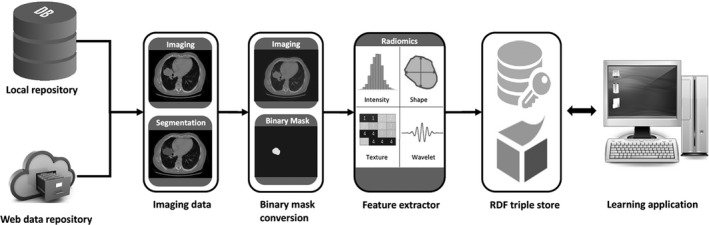
The generalized workflow of O‐RAW. First, DICOM imaging data are captured from a local or web repository. Second, DICOM images and the associated RTSTRUCT files are converted into binary masks according the feature extractor input requirements. The radiomics extractor then calculates features and exports these in a (usually) custom output format. The features are then mapped to RDF to achieve semantic interoperability and published in an http‐accessible endpoint, from which they can be queried with SPARQL and used in a learning application. O‐RAW, Ontology‐guided Radiomics Analysis Workflow; RDF, resource description framework.

Figure 1 shows the workflow of radiomics analysis proposed in O‐RAW. First, imaging data from a local repository or web data repository (i.e., XNAT) are retrieved (pyxnat library^26^). Second, PyRadiomics Extension takes standard DICOM‐RT inputs (DICOM images and the associated RTSTRUCT file) and parses them as arrays of voxel intensities and a binary mask for each volume of interest (VOI). Next, the arrays are passed into PyRadiomics that performs the above mentioned feature extraction and returns a Python dictionary object to PyRadiomics Extension. Then, PyRadiomics Extension parses the dictionary as a W3C‐compliant semantic web “triple store” (i.e., RDF) with metalabels attached from the radiation oncology ontology^27^ and the IBSI compliant Radiomics Ontology.^25^ The RDF result can be published to an http‐accessible endpoint, and examined via SPARQL Protocol and RDF Query Language (SPARQL) queries. The information stored in RDF format include the radiomics feature unique identifier, its name and value, pre‐processing approaches, VOI(s), the patient identifier, and the radiomics software used. Finally, an external application can perform machine learning algorithms on the RDF triple store and return results back to learning application. Briefly, PyRadiomics is the radiomics feature extractor, and PyRadiomics Extension is the input and output extension of PyRadiomics to handle DICOM images and RDF object. O‐RAW is the workflow incorporating these tools to make radiomics study easily and connect to external application. In principle this modular set‐up should allow for other modules e.g. other binary conversion methods (e.g. Plastimatch or CERR) or other radiomics feature extractor software (e.g. IBEX or CERR), if in‐ and output are known.

## 3 Results

In order to assess O‐RAW, three tests were performed in this study. In the first test, the ability of O‐RAW to handle multiple modalities was verified on four datasets, RIDER (CT), MMD (CT), CROSS (PET) and THUNDER (MR). The results show that O‐RAW can perform radiomics analysis on different imaging modalities. O‐RAW executed efficiently on these datasets on a laptop running Windows 7 operating system and 8GB RAM, on which the execution time was noted of a common radiomics analysis. This included DICOM images and associated RTSTRUCT matching, binary mask conversion, feature extraction (105 basic features), and conversion of RDF object. The results were (RIDER) 407.3, (MMD) 123.5, (CROSS) 513.2 and (THUNDER) 128.9 s for a single VOI.

**Figure 2:**
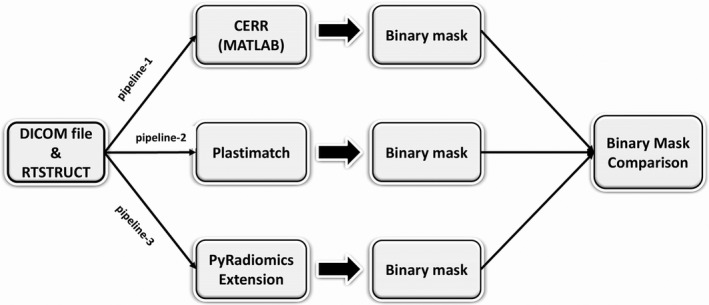
Three approaches (CERR, Plastimatch, and PyRadiomics Extension) shown in pipeline 1, 2, 3 were used for binary mask conversion. All three methods converted binary mask in NRRD format. One hundred randomly selected patients from MMD and CROSS datasets with CT and PET scans. The Dice similarity coefficient was used as the comparison measurement.

Second, to evaluate the method of binary mask conversion in O‐RAW, we used Plastimatch (version 1.7.3), PyRadiomics Extension and CERR (MATLAB) to convert binary masks for 100 randomly selected patients from MMD and CROSS datasets with CT and PET scans. This test led to a mask in NRRD format per VOI per application. The flowchart is shown in Fig. 2 (pipeline 1, 2 and 3). The Dice similarity coefficient^28^ of binary masks converted by three approaches were all exactly unity, which indicated that conversion by either Plastimatch, PyRadiomics Extension and CERR result in the same binary mask.

O‐RAW provides two user‐configurable options of output format, CSV (Comma delimited) and RDF (default). On the one hand, the simpler csv file output can be saved in the export directory given in the configuration file of O‐RAW, which contains the information of patient ID, VOI(s), and values of radiomic features. To track more details of radiomic features extraction (e.g., preprocessing methods), information should be saved in other flat tables, which is the limitation of using relational tables to present data using a rigid and predefined structure (known as a schema). Another and preferred option is to save radiomic features in the RDF format, which allows full expressivity of features and their details. The information can be retrieved by using SPARQL queries from a SPARQL endpoint (e.g., Blazegraph), which not only includes patient identifiers, VOI(s), and values of radiomic features, but also feature units, preprocessing and radiomics software details.

**Figure 3:**
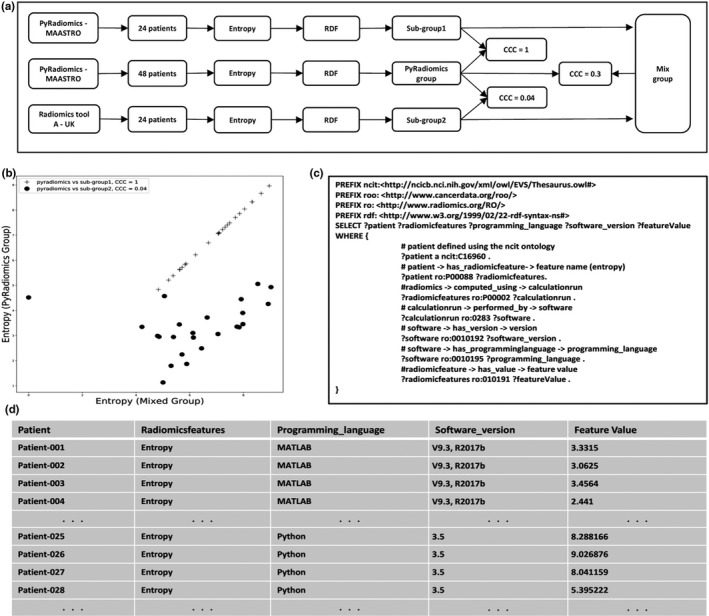
A real world example showing the use of an ontology‐supported description of a radiomics feature rather than just using the feature name. a) Flowchart indicating the use of two different radiomics feature extraction software implementation (PyRadiomics and MATLAB). b) Comparison of entropy values calculated by a mix of MATLAB and PyRadiomics (x‐axis) to PyRadiomics alone (y‐axis). The black dots indicate entropy calculated by MATLAB which has a concordance correlation coefficient of only 0.04 with PyRadiomics calculated entropy. c) SPARQL query to retrieve patient ID, radiomic feature name, programming language, software version, and feature value. d) Returned results of SPARQL query, where one can see the feature entropy was computed by two software implementations (MATLAB and Python/PyRadiomics).

In Fig. 3, we show a real world example, which demonstrates the importance of tracking computation details and the feasibility of describing radiomics via an RDF graph object. As shown in [Fig. 3(a)], a dataset with 48 patients is first randomly split into two subgroups without overlap, 24 patients for each. One subgroup data was sent to our partner in the UK. The radiomics of the subgroup1 were computed by PyRadiomics in MAASTRO and converted into RDF format. The radiomics of the subgroup2 were computed by Radiomics tool A and converted into RDF format as well. We combined radiomics of the two subgroups into a mixed group. Second, all 48 patients were computed by PyRadiomics only as PyRadiomics group. As a demonstration, we only select the feature entropy, which is defined identically, in terms of definition, formula and IBSI^29^ code, in both two software (PyRadiomics and Radiomics tool A) implementations. Concordance correlation coefficients (CCC) was calculated between the PyRadiomics group and mixed group, which yielded a CCC score of 0.3. The feature entropy values of two groups are shown in [Fig. 3(b)], where some cases in the mixed group were approximately identical to feature values in the PyRadiomics group, but some are not. Then, a simple SPARQL query shown in [Fig. 3(c)] was used to track the computation details. According to the returned result [Fig. 3(d)], the reason of the low CCC score is caused by using different software (PyRadiomics vs MATLAB radiomics toolbox). By analysing more computational details such as image preprocessing or filter methods, one could further investigate why these two entropy values are different between software implementation.

**Figure 4:**
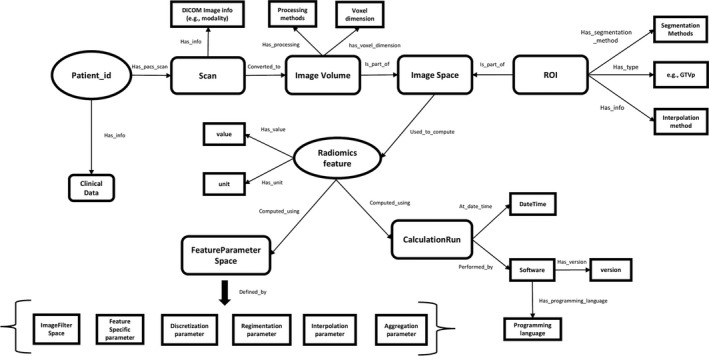
Visualization of nodes and relations as an RDF graph object generated by O‐RAW. The structure follows the IBSI compliant Radiomics Ontology. More details, see.^30^ The radiomics graph is able to link to a clinical data graph via Patient ID.

The visualization of RDF graph data generated by O‐RAW is presented in Fig. 4. This RDF graph object complies with the radiomics output structure of IBSI that as described in the Radiomics Ontology. Moreover, we showed an example in the Fig. S1, which describes how radiomic features are queried and computation details are tracked by a simple SPARQL query.

## 4 Discussion

Our work (O‐RAW) aims to address the lack of a standardized methodology for radiomics analysis. Most radiomics toolboxes are developed in‐house without public and standardized documentation on the details of the radiomics calculation and analysis, which makes it difficult to reproduce and validate radiomics studies. Besides in‐house developments, there are radiomics toolboxes which are publicly available, such as MITK,^21^ Mazda,^22^ PyRadiomics,^19^ IBEX,^23^ CERR^24^ and LifeX,^31^ and also commercial implementations exist. Each of these have different capabilities and limitations,^24^ showcasing the need for standardization efforts such as presented in this study.

The O‐RAW workflow package was developed using two open‐source component packages (PyRadiomics, and PyRadiomics Extension), of which functionalities are clarified here. PyRadiomics plays the role of a radiomics feature extractor using native file formats as input (e.g., NRRD format) and output (e.g., CSV). PyRadiomics Extension allows the use of standardized file formats as input (DICOM images and RTSTRUCT) and output (RDF) based on an ontology (i.e., Radiomics Ontology and Semantic DICOM ontology). The O‐RAW package integrates PyRadiomics and PyRadiomics Extension to implement batch processing including DICOM handling, ROI selection and exclusion, conversion from RDF to additional standardized file formats (CSV), and so on. The main innovation of O‐RAW is thus in orchestrating the workflow of a radiomics study. It can work with any radiomics feature extraction software, provided that they accept standard formats for input (i.e., file formats that can be read by ITK) and export data according to the Radiomics Ontology.

We selected PyRadiomics as the feature extractor in O‐RAW, as it best fits the concept of O‐RAW currently, in terms of well standardized documentation, universal programming language (Python), fully open‐sourced code, rapid maturation and an active community of user‐developers. With O‐RAW also fully open source, the process of making radiomic features FAIR using associated ontologies can be reproduced easily by others. Although we used PyRadiomics, it is important to note that the modular nature of O‐RAW allows easy integration of other radiomics toolkits as long as its input and output are known. When including a different radiomics toolkit, syntactic and semantic interoperability has to be created. With regard to syntactic interoperability, O‐RAW uses DICOM as its input syntax and RDF as its output syntax. In current radiomics feature extraction tools, either DICOM is already accepted (e.g., in CERR) requiring no change. If another standardized input format (e.g., NRRD) is accepted, then tools exist to convert DICOM into these formats (e.g., ITK). Output formats of radiomics toolkits vary (Python dictionary, CSV, etc) but many tools exist to convert such application data into RDF (https://www.w3.org/wiki/ConverterToRdf) – requiring simple configuration of those tools. Achieving syntactic interoperability is therefore not very difficult. With regard to semantic interoperability, recent standardization efforts for radiomic features, including the Radiomics Ontology, which has also implemented the IBSI standard, have emerged but have not yet been implemented widely. Custom code is thus necessary to map the native nomenclature to semantically standardized radiomic features. Also, details on how the radiomic features were calculated (e.g. filtering, software version etc.) need to be configured in O‐RAW. As an example, the current study used an in‐house MATLAB (Radiomics tool A) implementation from our UK partner and mapped its MATLAB output to RDF. In practice, this is a two‐step process: (a) Filling in the toolkit details in the configuration file of O‐RAW including computational details as described in the literature.^32^ This configuration table is used to create a base RDF graph containing information on the radiomics toolkit and its settings. (b) Mapping native names of features to Radiomics Ontology codes, by filling in mapping table with two columns: one is the feature names output from the users’ radiomics toolkit and the other one is the Radiomics Ontology codes. This mapping table is then used to create the individual radiomics features in RDF. The two extra steps can be avoided when using PyRadiomics, as the process of mapping is implemented automatically via the PyRadiomics Extension in O‐RAW.

In all cases, O‐RAW allowed users to track the details (e.g., feature calculation approaches or parameters) of each step within a typical radiomics analysis workflow. We feel the primary aim and benefit of O‐RAW was thus demonstrated which is to support reproducible and interoperable radiomics research with the use of ontologies, promote multicenter collaboration and hence increase the potential for wider external validation studies of radiomics‐assisted clinical prediction models.

The use of a standard and publicly accessible lexicon, the IBSI compliant Radiomics Ontology,^25^ explicitly documents the definitions and mathematical formulas of radiomic features. Using an ontology is a major improvement over using a human‐readable label alone, which is not sufficient to guarantee semantic equivalence and interoperability. We feel creating semantic interoperability through the use of ontologies is essential for the comparison and validation of radiomic studies, given the diverse software implementations, preprocessing approaches and feature labels which are in active use.

For example, using an ontology first forces one to choose if a feature called “entropy” is the Intensity Histogram Entropy (IBSI ID = TLU2)^25^ or textural feature Joint Entropy (IBSI ID = TU9B).^25^ Second, using an ontology, two users who compute Joint Entropy can also note what pixel spacing and software implementation was used to compute their respective Joint Entropies. We have shown in this study that O‐RAW can offer such detailed expressiveness by using the Radiomics Ontology to describe features and attach metadata for those features, resulting in semantic interoperability and ultimately FAIR data.

Finally, flat tables for radiomics output do not sufficiently capture the methodological steps that affect feature values. For instance, image resampling prior to features extraction might affect the result.^33^, ^34^ However, a two dimensional table that only describes the radiomic feature names and their values is not sufficient to determine which methodologies of pre‐ and postprocessing are used for radiomics calculation. It is impossible to know if radiomic features from two flat tables use the same pre‐processing method(s) without any further information, though their value might be equal.

We identified the challenges for radiomics research as: (a) lack of standardized methodology for radiomics analyses, (b) lack of a universal lexicon to denote features that are semantically equivalent, and (c) lists of feature values alone do not sufficiently capture the details of feature extraction that might nonetheless strongly affect feature values (e.g. image normalization or interpolation parameters). We have demonstrated that O‐RAW is capable of handling these challenges. First, the radiomics extractor used in O‐RAW is PyRadiomics, which is the largest open‐source radiomics package and has attracted more and more attention of researchers in the radiomics community. Second, the IBSI compliant Radiomics Ontology is applied in O‐RAW to guide radiomics analysis, which offers unambiguous metadata to note if two radiomic features are equivalent semantically or not. Third, the default output format is RDF within O‐RAW, which can link radiomic features and values to related meta‐data, such as patient ID, VOI, unit, preprocessing, and software version. The uniqueness of a radiomic feature is not the name of the feature, but the details describing how the feature is computed. As shown in Fig. 4, when two features have an identical name, they may not be identical. Their computation settings, such as image processing and filter methods, may be different, which can be tracked by using ontologies such as proposed in O‐RAW.

One of the benefits of using RDF graphs to store radiomics data which we will study in future work, that is it allows the linkage to other RDF graphs e.g. containing clinical data such as histology and other biological characteristics of the tumor and outcomes which are important in many radiomics studies to derive relations between the imaging phenotype and tumor genotype and to make clinically relevant prediction models. Similarly, RDF allows as to leverage work done in the Semantic DICOM ontology (https://www.ncbi.nlm.nih.gov/pubmed/25160167) and store the complete DICOM header in RDF and link it to the Radiomics RDF. This is part of our future works as it would make it unnecessary to derive and store DICOM header information (e.g. slice thickness and pixel spacing) in O‐RAW as is done now.

Finally, O‐RAW is able to generate FAIR data: (a) radiomics data and extraction details could be published with a Findable(F) and unique identifier; (b) radiomics data and metadata are described with the Radiomics Ontology, which make them accessible(A) and understandable by machines and humans; (c) data uses a formal, standardized and applicable ontology for knowledge representation, which makes interoperability(I) among multicenters possible; (d) data offers explicit information on provenance and licenses for reuse (R).

A current limitation of the O‐RAW package is that multicenter studies based on querying the feature RDFs must be based on PyRadiomics or converted to RDF triples. Future work will involve three aspects. First, we will to extend our method to convert features generated in native format and nomenclature into IBSI compliant, ontology‐based to other radiomics software, and will cooperate with other willing developers to address the challenge to create syntactic and semantic interoperability between radiomics studies. With such interoperability O‐RAW would allow identification of differences in feature calculation between different packages/vendors. Second, a distributed learning study among multicenters will be performed to link clinical outcome, DICOM header and radiomic features via O‐RAW. Finally, accurate and robust automatic segmentation of tumor tool will be integrated into our workflow. It means that O‐RAW will extract radiomics from original DICOM images without a requirement for any other (manual) annotation information (i.e., RTSTRUCT).

## 5 Conclusions

In this study, we successfully implemented O‐RAW for radiomics analysis from radiotherapy‐based images to ontology guided FAIR data. Its practical use and flexibility can greatly promote the advance of radiomics research and may help the associated achievements transfer to clinical practice. The development goal of O‐RAW is to help radiomics users on both input and output sides. First, it allows to import original DICOM images and RTSTRUCT files that are commonly used in the radiation oncology field. Second, the output is machine‐readable data with related ontologies, which promotes the standardization in terms of radiomic features, pre‐ and postprocessing.

## Conflict of interest

The authors have no conflict of interests to declare.

## Supporting information


**Fig S1:** Shows an example, which describes how radiomic features are queried and computation details are tracked by a simple SPARQL query. The radiomic features could be linked to the clinical data of the patient by patient ID.

**Table S1:** The primary characteristics of publicly available open‐source radiomics extraction tools.
