# Supplementary figures and images for: Technical Note: Ontology‐guided radiomics analysis workflow (O‐RAW)
Source: Med Phys. 2019 Oct 25;46(12):5677–84. doi: 10.1002/mp.13844 (PMC6916323; doi:10.1002/mp.13844)

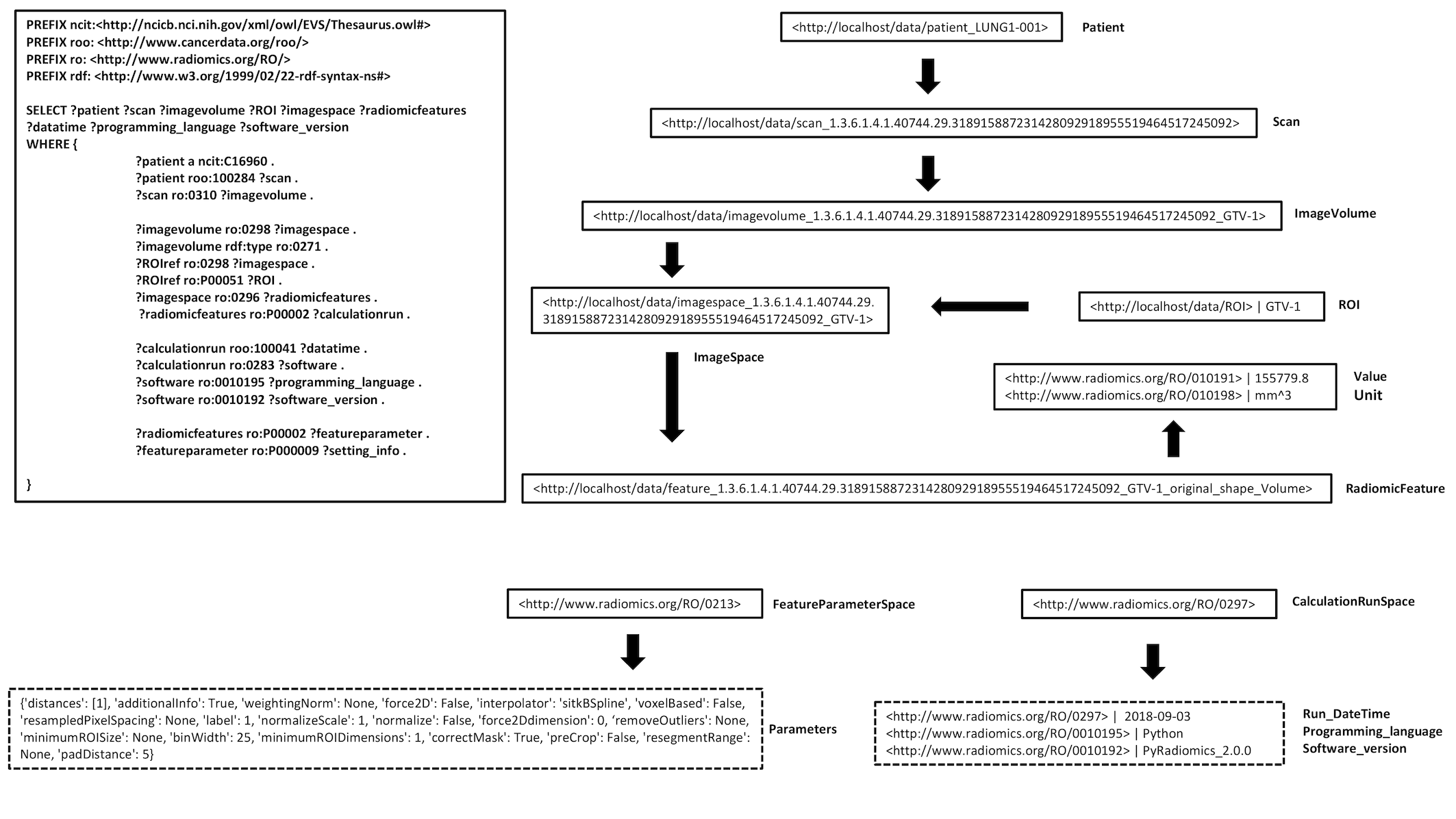

Supplement: Supplementary file 1 — Fig S1: Shows an example, which describes how radiomic features are queried and computation details are tracked by a simple SPARQL query. The radiomic features could be linked to the clinical data of the patient by patient ID. [file MP-46-5677-s001.tif]
