# Supplementary material for: Technical Note: Ontology‐guided radiomics analysis workflow (O‐RAW)
Source: Med Phys. 2019 Oct 25;46(12):5677–84. doi: 10.1002/mp.13844 (PMC6916323; doi:10.1002/mp.13844)
Supplement: Supplementary file 2 — Table S1: The primary characteristics of publicly available open‐source radiomics extraction tools. [file MP-46-5677-s002.doc]

Table 1: The primary characteristics of publicly available open-source radiomics extraction tools [1].

|  | **Programming language** | **IBSI feature definitions** | **Full OS compatibility** | **DICOM-RT**  **Import** | **Integrated visualization** | **Radiomics metadata storage** | **Built-in segmentation** | **Radiomics maps** |
| --- | --- | --- | --- | --- | --- | --- | --- | --- |
| **ITK** | C++ | No | Yes | Yes | No | No | No | Yes |
| **MaZda** | C++/Delphi | No | No | No | Yes | No | Yes | Yes |
| **PyRadiomics** | Python | Yes | Yes | No | No | No | No | No |
| **IBEX** | Matlab/C++ | No | No | Yes | Yes | Yes | Yes | No |
| **CERR** | Matlab | Yes | Yes | Yes | Yes | Yes | Yes | Yes |
